# Tooth Decay Promotes Senescence in Dental Pulp Stem Cells, Modifying Their Biological and Proteomic Profiles

**DOI:** 10.1002/jcp.70172

**Published:** 2026-04-15

**Authors:** Sebahat Melike Durukan, Mustafa Burak Acar, Banu Çiçek Tez, Ahmet Şimşek, Sura Hilal Ahmed Al‐Sammarrie, Zeynep Günaydın, Melis Güzel, Başak Bilge Süer, Şerife Ayaz Güner, Hüseyin Güner, Nicola Alessio, Kemal Erdem Başaran, Zeynep Burçin Gönen, Servet Özcan

**Affiliations:** ^1^ Genome and Stem Cell Center (GENKOK) Erciyes University Kayseri Türkiye; ^2^ Institute of Public Health, Epidemiology Charité—Universitätsmedizin Berlin Berlin Germany; ^3^ Department of Biology, Faculty of Science Erciyes University Kayseri Türkiye; ^4^ Department of Experimental Medicine Luigi Vanvitelli Campania University Naples Italy; ^5^ Deparment of Pediatric Dentistry, Faculty of Dentistry Ankara Medipol University Ankara Türkiye; ^6^ College of Applied Sciences, Department of Biotechnology University of Samarra Samarra Iraq; ^7^ LÖSANTE Hospital Ankara Türkiye; ^8^ Faculty of Health Sciences Nuh Naci Yazgan University Kayseri Türkiye; ^9^ Department of Molecular Biology and Genetics Izmir Institute of Technology İzmir Türkiye; ^10^ İzmir International Biomedicine and Genome Institute Dokuz Eylül University İzmir Türkiye; ^11^ İzmir Biomedicine and Genome Center (IBG) İzmir Türkiye; ^12^ Department of Molecular Biology and Genetics, Faculty of Life and Natural Science Abdullah Gül University Kayseri Turkey; ^13^ Department of Physiology, Faculty of Medicine Erciyes University Kayseri Türkiye; ^14^ Department of Oral and Maxillofacial Surgery, Faculty of Dentistry Erciyes University Kayseri Türkiye

**Keywords:** dental caries, DPSC, senescence, tooth decay

## Abstract

Dental caries is a prevalent oral health problem that significantly reduces an individual's quality of life; although, it can be effectively managed through restorative treatments. Even in cases where the caries does not reach the pulp, released microbial products from the lesion can still penetrate the pulp chamber, potentially inducing stress on pulp cells. In this study, we conducted a comparative analysis of the biological and proteomic profiles of dental pulp stem cells (DPSCs) isolated from clinically asymptomatic teeth with dentinal caries that had not reached the pulp and isolated from healthy teeth. Following biological evaluations, we examined proteomes of these DPSCs by conducting a shotgun proteomics approach. Our findings show that DPSCs from decayed teeth exhibit a significantly higher proportion of senescent cells. Proteomic profiling revealed upregulation of inflammatory signaling, extracellular matrix remodeling, and senescence‐associated secretory phenotype (SASP) related proteins. Additionally, we observed an upregulation in the expression of proteins associated with extracellular matrix (ECM) remodeling and components of the SASP, which are hallmarks of the senescence process. The study reveals that DPSCs can be affected by stress from carious lesions, even when the pulp appears clinically intact. Senescence and inflammatory response in these affected cells may have deleterious effects on other tissues within the organism. Consequently, restorative treatments should consider targeting not only the decayed tissue but also the senescent cells within the pulp that may have been affected by the stress induced by caries.

## 1 Introduction

The dental pulp consists of odontoblasts, fibroblasts, immune system cells, and undifferentiated stem cells. Dental pulp stem cells (DPSCs) reside in the perivascular niche of this tissue, which is surrounded by dentin and exists within a connective tissue structure (Ledesma‐Martínez et al. 2016). In addition to maintaining pulp homeostasis, DPSCs are widely used in regenerative research due to their accessibility, high proliferation capacity, and differentiation potential. Their strong paracrine activity allows them to exert effects beyond the local environment (Bousnaki et al. 2022).

Despite being enclosed by mineralized dentin, pulp tissue and DPSCs are exposed to stressors such as microbial products and inflammatory mediators (Fawzy El‐Sayed et al. 2019). Among these, dental caries is one of the most common stress factors, arising from interactions between oral bacteria, fermentable carbohydrates, and host factors like saliva (Selwitz et al. 2007). As caries advances, microbial invasion of dentin elicits inflammatory responses in the pulp (Farges et al. 2015), leading to pulpitis. If untreated, this can result in irreversible damage and early tooth loss. In reversible cases, DPSCs play a central role in regeneration, particularly via tertiary dentin formation, though they may also contribute to degeneration (Rosa et al. 2011; Ricucci et al. 2014). Their ability to differentiate into odontoblasts is crucial for sealing off lesions and preserving pulp vitality. However, exposure to stress can compromise this capacity. The biological status of DPSCs is a key determinant of their regenerative potential, and thus understanding their response to carious environments is critical. While conventional treatments restore tooth structure, they do not necessarily preserve or restore pulp function. Therefore, it is crucial to investigate how dentinal caries that do not extend into the pulp chamber affect the molecular profile of DPSCs.

Microbial products penetrating dentin, especially lipopolysaccharides (LPS) from gram‐negative bacteria, are well‐known activators of Toll‐like receptor 4 (TLR4)‐mediated inflammatory signaling, leading to both intracellular stress and the release of pro‐inflammatory cytokines (Feng et al. 2018). This chronic or repeated stimulation not only disrupts tissue homeostasis but may also drive long‐term cellular responses beyond inflammation. We hypothesize that one such response is the induction of cellular senescence in DPSCs. Even in cases where the pulp is not visibly compromised, the proximity of microbial activity may be sufficient to trigger senescence‐associated responses. These senescent cells, in turn, exhibit reduced regenerative capacity and can actively sustain inflammation through the secretion of senescence‐associated secretory phenotype (SASP) factors.

In this study, we analyzed pulp tissues from healthy teeth and teeth with carious lesions that did not penetrate the pulp chamber. We assessed stem cell markers, senescence, and apoptosis, and conducted a bottom‐up proteomic analysis to explore how intracellular pathways and secretory profiles are altered. Bioinformatic analysis revealed that dental caries activates pathways related to inflammation and senescence in DPSCs. These findings highlight how microbial stress compromises pulp regenerative function, offering insights critical for the development of new therapeutic strategies that aimed at preserving or restoring pulp vitality beyond morphological repair.

## 2 Materials and Methods

### 2.1 Isolation and Characterization of DPSC from Healthy and Decayed Teeth (Caries‐Affected Teeth)

**Figure 1:**
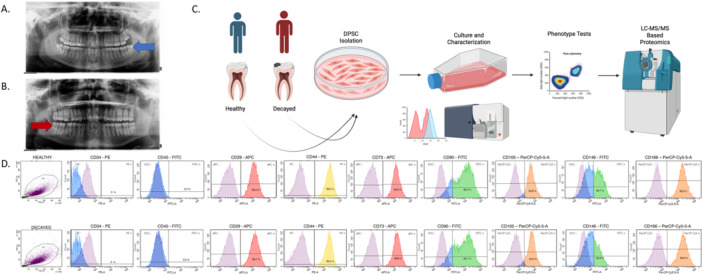
(A) Mandibular right semi‐impacted third molar (tooth #38) with healthy pulp tissue, (B) Mandibular left semi‐impacted third molar (tooth #48) presenting with asymptomatic dentinal caries. (C) Experimental design. (D) Analysis of cell surface markers of Healthy and Decayed DPSC via flow cytometry. This figure illustrates the characterization of dental pulp stem cells (DPSCs) derived from healthy and carious teeth using flow cytometry. The analysis confirms the absence of hematopoietic lineage markers CD34 and CD45 in both cell populations, while demonstrating the positive expression of mesenchymal stem cell markers CD29, CD44, CD73, CD90, CD105, and CD166. Additionally, differential expression levels of CD146, a cell adhesion glycoprotein, are presented between the two groups.

DPSCs were obtained from the extracted third molar teeth of healthy donors aged 18–40, with the approval of the Erciyes University Clinical Research Ethics Committee (Approval No: 2019/573). The study has been carried out in accordance with the World Medical Association Declaration of Helsinki and that all subjects provided written informed consent. Tissue samples from eight different patients were collected from the healthy pulp (HP) group. Patients' samples were obtained from semi‐impacted third molars, and extractions were recommended for patients with impacted teeth according to the position of the existing teeth in the jaw, and the maxillary and mandibular arches were not differentiated. Affected pulp (AP) tissue samples from eight different patients' teeth with deep dentin caries were obtained. The patients' samples were taken from their third molar teeth, with an indication for extraction based on their clinical findings. Pulp tissue samples classified as affected pulp were obtained from the third molar teeth of eight different patients presenting with deep dentinal caries, but without clinical or radiographic signs of irreversible pulpitis. All teeth were asymptomatic, showing no evidence of spontaneous pain, prolonged sensitivity, swelling, or periapical pathology **(**Figure 1A **)**. These findings suggest a subclinical inflammatory state of the pulp without direct pulpal exposure. The extractions were performed based on clinical or orthodontic indications.

Pulps were extracted with sterile clamps and were chopped into small pieces. Samples were digested via collagenase (Type II, 125 U/ml) (Sigma‐Aldrich, St. Louis, MO, USA) for approximately 45 min at 37°C thermal block. Cells were washed twice with Dulbecco's Phosphate‐Buffered Saline (DPBS) (Ca, Mg‐free) (Gibco, Waltham, MA, USA) after the digestion process. Cell pellet was resuspended at 10% Fetal Bovine Serum (FBS) (FBS; Gibco, Waltham, MA, USA), 25 ng/mL l‐Ascorbic Acid (Sigma‐Aldrich, St. Louis, MO, USA), 4 ng/mL dexamethasone (Sigma‐Aldrich, St. Louis, MO, USA), 5 ng/mL basic Fibroblast Growth Factor (b‐FGF) (Gibco, Waltham, MA, USA), and 10 ng/mL Epidermal Growth Factor (EGF) (Gibco, Waltham, MA, USA) containing Alpha Minimum Essential Medium (α‐MEM) (with nucleosides) (Gibco, Waltham, MA, USA) and was cultured at 37°C. For subsequent experiments, equal number of cells (5×10^5^) derived from different donors within the decayed and healthy groups were pooled separately at the end of passage 1. To characterize the isolated stem cells, cluster of differentiation 29 (CD29), cluster of differentiation 44 (CD44), cluster of differentiation 90 (CD90), cluster of differentiation 73 (CD73), cluster of differentiation 105 (CD105), and cluster of differentiation 146 (CD146), cluster of differentiation 166 (CD166), surface antigens were used as positive markers, and cluster of differentiation 34 (CD34) and cluster of differentiation 45 (CD45) (BD Biosciences, Franklin Lakes, NJ, USA) were used as negative markers. Characterization was completed by using BD FACS Aria III flow cytometry (BD Biosciences, Franklin Lakes, NJ, USA), and analysis was performed via BD FACS Diva 8.0.1 software (BD Biosciences, Franklin Lakes, NJ, USA).

### 2.2 Senescence Associated β‐Galactosidase Assay and C_12_FDG Quantitative Senescence Assay

The percentage of senescent cells in the Mesenchymal Stem Cells (MSCs) population was analyzed by both Senescence‐Associated β‐Galactosidase (SA‐β‐Gal) Assay and 5‐dodecanoylaminofluorescein di‐β‐d‐galactopyranoside (C_12_FDG) assay. To perform‐ SA‐β‐Gal Assay, cultured cells were fixed with 0.2% Glutaraldehyde solution (Sigma‐Aldrich, St. Louis, MO, USA). Then samples were washed twice and incubated with a staining solution that contains 40 mg/mL 5‐bromo‐4‐chloro‐3‐indolyl β‐d‐galactopyranoside (X‐Gal) (Sigma‐Aldrich, St. Louis, MO, USA) overnight. After incubation, cells were washed to discard the staining solution, and blue‐stained cells were counted according to the previously described protocol (Debacq‐Chainiaux et al. 2009). To measure the senescent cell percentage quantitatively, the C_12_FDG assay was performed. The medium of cultured cells was alkylated by the addition of Bafilomycin (Sigma‐Aldrich, St. Louis, MO, USA) A1 at 100 nM final concentration. After bafilomycin‐containing media were discarded, 20 mM C_12_FDG (Thermo Fisher Scientific, Waltham, MA, USA) solution was added to the cells and incubated for 2 h at 37°C. Following the incubation, cells were detached by trypsinization and resuspended in 500 µL of cell wash. Analysis was done by using BD FACSAria™ III Flow Cytometer and BD FACSDiva™ version 8.0.1 software.

### 2.3 Annexin V – 7‐AAD Apoptosis Assay

Apoptotic and dead cell ratios were measured with Annexin V and 7‐Aminoactinomycin D (7‐AAD) assay. Detached cells were washed and resuspended in 100 µL Annexin V binding buffer (BioLegend, San Diego, CA, USA). 5 µL Fluorescein Isothiocyanate (FITC)‐conjugated Annexin V (BioLegend, San Diego, CA, USA) and 5 µL 7‐AAD (BD Biosciences, San Jose, CA, USA) viability staining solution added on samples. Cells were incubated for 15 min at room temperature. After incubation, we added 400 µL Annexin V binding buffer to each tube and analyzed the samples by using BD FACSAria™ III Flow Cytometer and BD FACSDiva™ version 8.0.1 software.

### 2.4 Cell Cycle Assay

To determine the cell cycle distribution of cells, a 7‐AAD‐based cell cycle assay was performed. Cells were harvested and fixed with ice‐cold 70% ethanol. After 2‐h incubation at −20°C, fixed cells were washed twice with cell wash buffer and resuspended in 100 µL cell wash buffer. 10 µL 7‐AAD solution was added to the tubes. Cell Cycle analysis was completed by using BD FACS Aria III flow cytometry and BD FACS Diva 8.0.1 software.

### 2.5 Immunoflorescent Staining and Morphometric Analysis

Immunofluorescence (IF) staining was performed to evaluate the expression levels of cellular senescence markers p16 and p21, as well as inflammatory signaling pathway proteins Nuclear Factor kappa B (NF‐κB), Interleukin‐1 beta (IL‐1β), Interleukin‐6 (IL‐6), and Tumor Necrosis Factor alpha (TNF‐α). Cells were cultured on glass coverslips (Thermo Fisher Scientific, Waltham, MA, USA), fixed with 4% paraformaldehyde, permeabilized using 0.1% Triton X‐100 (Sigma‐Aldrich, St. Louis, MO, USA), and incubated with a blocking solution, 0.5% Bovine Serum Albumin (BSA) (BSA, Sigma‐Aldrich, St. Louis, MO, USA) and 22.52 mg/mL glycine (Sigma‐Aldrich, St. Louis, MO, USA) containing Phosphate‐Buffered Saline with Tween−20 (PBST) (PBST, Gibco, Thermo Fisher Scientific, Waltham, MA, USA) buffer to prevent non‐specific binding.

Cells were then incubated overnight at 4°C with primary antibodies. The following antibodies were used: mouse monoclonal anti‐IL‐1β (1:500) (Thermo Fisher Scientific, Waltham, MA, USA), mouse anti‐IL‐6 (1:500) (Thermo Fisher Scientific, Waltham, MA, USA), rabbit anti‐TNF‐α (1:500) (Thermo Fisher Scientific, Waltham, MA, USA), rabbit anti‐NF‐κB (1:400) (Cell Signaling Technology, Danvers, MA, USA), anti‐p21 antibody (1:500) (Abcam, Cambridge, UK), and rabbit anti‐p16 antibody (1:100) (Abcam, Cambridge, UK). To assess cytoskeletal integrity and serve as a loading/control marker, β‐actin expression was simultaneously detected using rabbit‐ or mouse‐derived anti‐β‐actin antibodies at dilutions of 1:200 and 1:500 (Abcam, Cambridge, UK), respectively.

Following primary antibody incubation, cells were incubated for 1 h at room temperature with species‐specific secondary antibodies: Alexa Fluor 594–conjugated anti‐mouse or anti‐rabbit antibodies (1:500) (Invitrogen, Thermo Fisher Scientific, Waltham, MA, USA) and Alexa Fluor 488–conjugated anti‐rabbit or anti‐mouse antibodies (1:500) (Invitrogen, Thermo Fisher Scientific, Waltham, MA, USA). Nuclei were counterstained with 4′,6‐diamidino‐2‐phenylindole (DAPI) (Thermo Fisher Scientific, Waltham, MA, USA). Coverslips were mounted using an antifade mounting medium and images were acquired using a fluorescence microscope. Protein expression levels, nucleus area and cytoplasm area were quantitatively analyzed based on fluorescence intensity measurements by using ImageJ software.

### 2.6 Whole Cell Proteome Sample Preparation

To prepare whole cell samples for mass spec analysis, the previously described method by Kulak and colleagues (2014) was used (Kulak et al. 2014). Accordingly, 20 µL lysis buffer (6 M Guanidine Hydrochloride (Sigma‐Aldrich, St. Louis, MO, USA), 10 mM Tris(2‐carboxyethyl)phosphine (Sigma‐Aldrich, St. Louis, MO, USA), 40 mM 2‐Chloroacetamide (Sigma‐Aldrich, St. Louis, MO, USA), 100 mM Tris‐HCl pH 8.5) was added to 2 × 10^5^ cells were boiled for 5 min. After boiling, samples were cooled by incubation on ice, and samples were sonicated for 15 min in an ultrasonic water bath. To remove the cell debris, such as DNA, RNA, or membranous parts, cells were centrifuged at 20000 x *g* for 15 min at +4°C. The obtained lysate was added to the Styrenedivinylbenzene‐Reverse Phase Sulfonate (SDB‐RPS) StageTips. 280 ng Lysyl Endopeptidase (Promega, Madison, WI, USA) containing 40 µL of dilution solution (25 mM Tris‐HCl pH:8.5, 10% Acetonitrile (Sigma‐Aldrich, St. Louis, MO, USA)) was added to each sample and after overnight incubation at 37°C. After Lys‐C digestion, 800 ng Trypsin (Promega, Madison, WI, USA) containing 140 µL solution was added to StageTips and incubated for 4 h. Digested peptides were acidified and loaded onto SDB‐RPS disks with loading buffer [2% Trifluoroacetic Acid (Sigma‐Aldrich, St. Louis, MO, USA)]. Peptide fragments were washed at least three times with wash buffer (0,2% TFA) and were eluted from StageTips with a gradually increased percentage of ACN. Samples were dried in a vacuum centrifuge (Eppendorf, Hamburg, Germany). To analyze the samples, resuspension solution (5% ACN and 0.1% Formic Acid (Sigma‐Aldrich, St. Louis, MO, USA)) was added to the samples just before Liquid Chromatography–Tandem Mass Spectrometry (LC‐MS/MS) analysis.

### 2.7 Secretome Sample Preparation

To obtain conditioned media, the cell culture medium was replaced with serum‐free medium when cells reached approximately 70% confluence, and the cells were incubated for 24 h. Conditioned medium was collected into Falcon tubes (Corning, Corning, NY, USA) from each group, and debris was discarded by centrifugation at 10,000 x *g* for 10 min. StrataClean Beads (Agilent Technologies, Santa Clara, CA, USA) were added to conditioned media and incubated overnight at +4°C. Following the incubation step, the beads were washed twice with Tris–Ethylenediaminetetraacetic Acid (TE) buffer (50 mM Tris, 10 mM Ethylenediaminetetraacetic Acid (EDTA), pH 7) and dried with SpeedVac (Eppendorf, Hamburg, Germany). 2% (w/v) RapiGest™ Surfactant (RapiGest) (Waters/Agilent, USA) and Triethylammonium Bicarbonate (TEAB) (Sigma‐Aldrich, St. Louis, MO, USA). 20 mM Tris(2‐carboxyethyl)phosphine (TCEP) (Sigma‐Aldrich, St. Louis, MO, USA) containing resuspension buffer was added to beads, and samples were incubated at 60°C for 30 min. Alkylation of cysteine residues was done by incubation with Iodoacetamide (IAA) (IAA, Bio‐Rad, Hercules, CA, USA) RT for 15 min. Proteolytic digestion was performed by using 200 ng Lys‐C (Promega, Madison, WI, USA) and 800 ng Trypsin‐Gold (Promega, Madison, WI, USA), respectively. Protein samples were incubated for 4 h with Lys‐C and overnight with trypsin at 37°C. Beads were removed from digested peptides by centrifugation at 10,000 g for 1 min, and supernatants were collected into new tubes. Digested peptides were acidified with 1% trifluoroacetic acid (Thermo Fisher Scientific, Waltham, MA, USA). C18 reversed‐phase disks (Empore™ C18, St. Paul, MN, USA) were activated with methanol, washed with elution buffer 0.1% Acetic Acid, 80% ACN), and equilibrated with wash buffer 0.1% Acetic Acid). Acidified samples were loaded into tips and washed twice with wash buffer. After the washing step, digested peptides were eluted with 0.1% Acetic Acid, 80% ACN containing elution buffer. Peptides were dried with SpeedVac and stored until Liquid Chromatography/Mass Spectrometry (LC/MS) analysis.

### 2.8 LC‐MS/MS Analysis

Mass spectrometry analysis was performed via LC‐MS/MS Eksigent ekspert™ nanoLC 400 System (AB SCIEX, Framingham, MA, USA) and AB Sciex Triple ToF 5600+ (AB SCIEX, Framingham, MA, USA). A MonoCap 250 mm C18 (GL Sciences, Tokyo, Japan) reverse phase column was used in the trap‐elute mode with 180‐min gradients for the separation of peptide fragments. 4%–40% ACN gradient was used during the separation process. Data‐dependent acquisition (DDA) tandem mass spectrometry (MS/MS) analysis of peptides was performed after charging the molecules via electrospray ionization (ESI) with the 2400 V and 75°C interface heater temperature (IHT) parameters.

Analysis of generated data by instrument and analytical data measurements were completed with Analyst® TF v.1.6 (AB SCIEX, Framingham, MA, USA). Peptides and product‐ions quality were evaluated with PeakView (AB SCIEX, Framingham, MA, USA). The raw data in.wiff format, which contains mass‐to‐charge (m/z) values, was analyzed by Protein Pilot 4.5 Beta (AB SCIEX, Framingham, MA, USA), and false discovery rate (FDR) analysis was performed in order to assure the identification quality.

### 2.9 Label Free Quantification (LFQ) Analysis

Label free quantification (LFQ) analysis of data was done by using MaxQuant software (version 1.6.14.0; Max Planck Institute of Biochemistry, Martinsried, Germany; https://www.maxquant.org). Mass tolerance was determined as 20 ppm, and MS/MS mass tolerance was 0.05 Da. Carbamidomethyl was used as a fixed modification, whereas acetylation and oxidation were used as variable modifications. Quantitative differences between groups were analyzed via Linear Models for Microarray Data (Limma; R/Bioconductor, https://bioconductor.org/packages/release/bioc/html/limma.html), which is an R/Bioconductor software package (https://bioconductor.org/packages/release/bioc/ html/limma. html). Volcano plots and heatmap visualizations were generated with R (R version 4.0.2, R Foundation for Statistical Computing, Vienna, Austria), a kind of Limma software.

### 2.10 Bioinformatics and Biostatistics Evaluations

Statistical significance was determined with one‐way and two‐way analysis of variance (ANOVA) analysis. Data were analyzed with a GraphPad Prism statistical software package (GraphPad Software, San Diego, CA, USA). Gene Ontology Biological Process (GO‐BP) analysis was completed by using STRING database (STRING STRING Consortium, https://string-db.org). The obtained GO‐BP results were visualized by using R Studio. Furthermore, the Reactome Pathway Database (Reactome, EMBL‐EBI, Hinxton, UK) was used for pathway analysis, and Cytoscape (version 3.9.1; Cytoscape Consortium, San Diego, CA, USA) was used for the visualization of pathways and associated proteins. Investigation of the obtained proteome data was completed via Ingenuity Pathway Analysis (IPA) (IPA; QIAGEN, Hilden, Germany). Canonical pathways and disease and function annotation were performed with generated data sets.

## 3 Results

In order to compare molecular and phenotypic differences of DPSCs from healthy and carious teeth, we conducted biological and proteomic evaluations. DPSCs obtained from healthy and caries‐affected teeth were pooled after primary culture to avoid individual variations between samples. Commonly applied Mesenchymal Stem Cell markers (CD34, CD45 negative, and CD29, CD44, CD73, 105 positive) were used for the characterization of DPSCs, also, CD146 and CD166 were added to the panel. Characterization results indicated that cultured cells were highly positive for all of the mesenchymal markers except CD146 The percentage of cells expressing CD146, a cell surface glycoprotein that plays a part in cell adhesion, was 33.3% in the caries group and 46.7% in the healthy group. Cells were found negative for haemopoietic markers CD34 and CD45 **(**Figure 1 **)**.

### 3.1 DPSCs of Caries‐Affected Teeth Exhibit Senescent Phenotype

**Figure 2:**
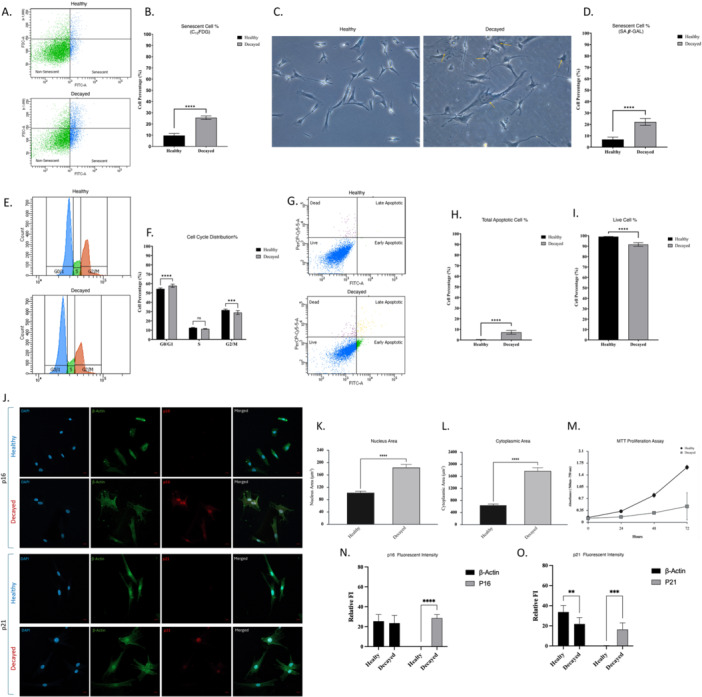
Phenotypic comparison of dental pulp stem cells (DPSCs) derived from healthy and carious teeth. (A) Flow cytometry analysis of the C_12_FDG assay reveals a higher proportion of senescent cells in DPSCs from carious teeth compared to those from healthy teeth. (B) Quantitative statistical analysis of C_12_FDG assay results. (C) Representative microscope pictures from the SA‐β‐Gal assay; senescent cells (characterized by enlarged and flattened morphology, increased nuclear size and multinucleation, elevated lysosomal content, and positive blue staining) are indicated by yellow arrows. (D) Statistical analysis of SA‐β‐Gal positive cell counts. (E) Cell cycle distribution analysis shows distinct differences between the two groups. (F) Statistical comparison of cell cycle phase distribution. (G) Annexin V/7‐AAD flow cytometry analysis indicates a slightly elevated apoptosis rate in DPSCs from carious teeth. (H, I) Statistical analyses of apoptosis and cell viability assay results. (J) Representative confocal microscopy images of Healthy and Decayed cells. Panels show staining for DAPI (blue; nuclei), β‐Actin (green; cytoskeleton), and senescence markers p16 or p21 (red). (K) Quantitative comparison of the nuclear surface area (μm²) between Healthy and Decayed cells. Decayed cells exhibit significantly enlarged nuclei, a hallmark of cellular senescence. (L) Measurement of the total cytoplasmic spread (μm2). Decayed cells show a markedly increased cell surface area compared to Healthy controls. (M) Growth curves showing cell viability and proliferation rates over a 72‐h period. The Decayed group demonstrates a significant arrest in proliferative capacity compared to the Healthy group. (N) Quantitative analysis of Relative Fluorescence Intensity for p16. A significant upregulation of p16 expression is observed in the Decayed group. (O) Comparison of Relative FI for β‐Actin and p21. The Decayed group shows a significant increase in p21 expression alongside a slight decrease in β‐Actin intensity.

C12FDG‐based quantitative senescence assay showed that the percentage of senescent cells was higher in DPSCs derived from the caries‐affected group compared to those from the healthy group. The senescent cell ratio was 9.70% ± 1.66 in healthy DPSCs, whereas DPSCs from carious teeth exhibited 25.60% ± 1.44 senescent cells **(**Figure 2A,B; Table 1A **)**. Appearance of DPSCs obtained from decayed tooth was showing senescent features with widened morphology, polynuclei, and increased lysosomal content **(**Figure 2C **)**. Consistent with these observations, the senescence‐associated β‐galactosidase (SA‐β‐gal) assay revealed a significantly higher proportion of senescent cells in the carious group (22.11% ± 2.88) compared with the healthy group (6.78% ± 1.87) **(**Figure 2D; Table 1B **)**. We observed an altered cell cycle distribution of DPSCs between the decayed and healthy groups. The cell percentage of decayed group was higher in G0/G1 phase with 57.73% (± 1.6), while the percentage of healthy DPSCs was 54.35% (± 0.88). We observed higher ratios in S and G2/M phase in the healthy DPSCs population, which were 12.5% (± 0.35) and 31.65% (± 0.88), respectively. Accordingly, lower S and G2/M ratios were observed in the decayed DPSCs population with 11.33% (± 0.33) and 28.98% (± 1.91), respectively. **(**Figure 2E,F **) (**Table 1C **)**. Annexin V and 7‐AAD assay revealed that there was an increase in the percentage of apoptotic cells in the decayed DPSCs population when compared with the healthy group, consequently, the percentage of live cells decreased in the decayed DPSCs population. We observed 7.25% (± 1.63) apoptotic cells and 91.53% (± 1.6) live cells in the decayed population. Whereas, in the healthy cell population, the total apoptotic cell percentage was 0.30% (± 0.16), and the live cell percentage was 99.08% (± 0.13) **(**Figure 2G–I **) (**Table 1D **)**. Furthermore, conducted quantitative morphometric analysis revealed significant alterations in cellular morphology between the experimental groups. DPSCs derived from carious teeth exhibited enlarged nuclei compared with healthy cells, with nuclear area increasing from 102.2 ± 4.69 µm² to 183.4 ± 10.27 µm². Similarly, cytoplasmic area increased markedly from 639.75 ± 41.82 µm² in healthy cells to 1775.3 ± 107.09 µm² in DPSCs derived from carious teeth (Figure 2J–L **) (**Tables 1E and 1F **)**. Assessment of proliferative capacity by using the MTT assay showed that cells derived from healthy teeth displayed higher proliferation rates, whereas cells obtained from carious teeth exhibited markedly reduced proliferative capacity **(**Figure 2M **)**. Finally, the expression levels of the senescence‐associated proteins p16 and p21 were evaluated by immunofluorescence staining. Both markers showed significantly increased expression in DPSCs derived from carious teeth compared with healthy cells. In healthy samples, p16 and p21 fluorescence signals were close to background levels, whereas strong staining was observed in the carious group. Quantitative analysis revealed p16 intensity of 28.55 ± 3.65 and p21 intensity of 16.29 ± 6.61 in DPSCs derived from carious teeth **(**Figure 2J,M,N **) (**Tables 1G and 1H **)**.

**Table 1:** Senescent and apoptotic cell percentages.

	Healthy					Decayed				
A	Senescent (*β*‐Gal +)					Senescent (*β*‐Gal +)				
	6,78% (± 1,87)					22,11% (± 2,88)				
	*p* < 0,0001					*p* < 0,0001				
B	Senescent (C_12_FDG +)					Senescent (C_12_FDG +)				
	9,70% (± 1,66)					25,60% (± 1,44)				
	*p* < 0,0001					*p* < 0,0001				
C	Live	Dead	Late apoptotic	Early apoptotic	Total apoptotic	Live	Dead	Late apoptotic	Early apoptotic	Total apoptotic
	99,08% (± 0,13)	0,63% (± 0,24)	0,25% (± 0,17)	0,05% (± 0,05)	0,30% (± 0,16)	91,53% (± 1,6)	1,23% (± 0,15)	1,13% (± 0,26)	6,12% (± 1,52)	7,25% (± 1,63)
	*p* < 0,0001				*p* < 0,0001	*p* < 0,0001				*p* < 0,0001
D	G0/1	S	G2/M			G0/1	S	G2/M		
	54,35% (± 0,88)	12,5% (± 0,35)	31,65% (± 0,88)			57,73% (± 1,6)	11,33% (± 0,33)	28,98% (± 1,91)		
	*p* < 0,0001	*p* = 0,0965	*p* = 0,0002			*p* < 0,0001	*p* = 0,0965	*p* = 0,0002		
E	Nucleus Area (µm^2^)					Nucleus Area (µm^2^)				
	102,2 (± 4,69)					183,4 (± 10,27)				
	*p* < 0,0001					*p* < 0,0001				
F	Cytoplasmic Area (µm^2^)					Cytoplasmic Area (µm^2^)				Nucleus Area (µm^2^)
	639,75 (± 41,82)					1775,3 (± 107,09)				183,4% (± 10,27)
	*p* < 0,0001					*p* < 0,0001				*p* < 0,0001
G	p16 Intensity					p16 Intensity				
	0,0 (± N/A)					28,55 (± 3,65)				
	*p* < 0,0001					*p* < 0,0001				
F	p21 Intensity					p21 Intensity				
	0,0 (± N/A)					16.29 (± 6,61)				
	*p* =0,0004					*p* = 0,0004				

*Note:* Cell distributions of each group. A. SA *β*‐Galactosidase Assay results. B. Quantitative senescence test C_12_FDG Assay Results. C. Annexin V/7‐AAD Assay results. D. Cell Cycle Assay results. E. Comparison of nucleus area between groups. F. Comparison of cytoplasm area between groups. G. Relative p16 Intensity. H. Relative p16 Intensity.

### 3.2 Comparative Proteomic Analysis of Unique Proteins for Each Group

**Figure 3:**
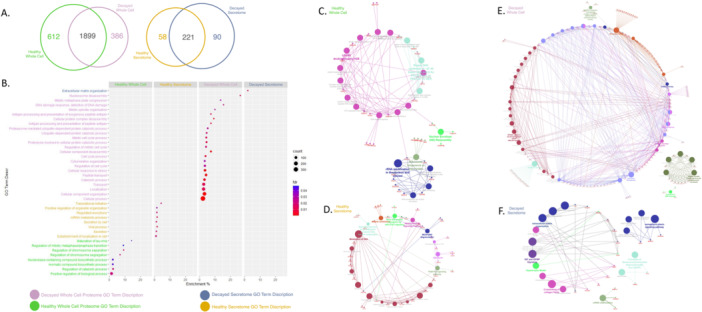
Comparative proteomic and pathway analysis of DPSCs derived from healthy and carious teeth. (A) Venn diagrams showing the number of proteins identified in the whole cell proteome and secretome samples for each group. (B) Gene Ontology (GO) Biological Process enrichment results are visualized as a bubble chart, highlighting prominent biological processes in carious tooth DPSCs, including extracellular matrix (ECM) remodeling, antigen presentation, and stress response. (C) Reactome pathway enrichment analysis of whole cell proteome from healthy tooth DPSCs. (D) Reactome pathway enrichment analysis of secretome proteins from healthy tooth DPSCs. (E) Reactome analysis of whole cell proteome from carious tooth DPSCs shows enrichment in NF‐κB signaling and cell cycle checkpoint pathways. (F) Reactome analysis of secretome proteins from carious tooth DPSCs reveals enrichment in IGF‐IGFBP signaling, ECM organization, and senescence‐associated secretory phenotype (SASP)‐related pathways.

According to data obtained from senescence, cell cycle, and apoptosis assays, we observed striking alterations in biological and phenotypic properties of decayed tooth DPSCs. Therefore, we followed an LC‐MS/MS‐based shotgun proteomic approach to find out the molecules, biological processes, and pathways behind the aforementioned phenotype alterations. Firstly, Venn analysis was performed, the number of unique proteins for each group was determined, and the accession numbers of these molecules were transformed to gene names and listed. Whole cell proteome analysis revealed 612 unique proteins for the healthy group and 386 unique proteins for the decayed group. 1899 proteins were found to be commonly expressed in both groups. We found 58 unique proteins for the healthy cells' secretome and 90 unique proteins for the decayed cells' secretome. In the secretome of these two groups, 221 proteins were found to be common **(**Figure 3A **)**. The identified unique proteins for each group were subjected to Gene Ontology Biological Process (GOBP) analysis. GO‐BP analysis of decayed tooth DPSCs revealed ontologies about cellular stress response, cell cycle regulation, and various catabolic processes. Additionally, we observed ontologies about antigen processing and presentation. Extracellular matrix organization was the only enriched ontology that has been found in GOBP analysis of decayed group secretome. While the decayed group cells were dealing with stress and antigen‐expressing associated molecules, GOBP analysis of healthy tooth DPSCs indicated regular metabolic and biological processes such as RNA metabolism, mitotic phase transition, chromosome organization, and secretion **(**Figure 3B **)**. Following Gene Ontology Biological Process enrichment analysis, we conducted pathway analysis by using the Reactome database. Consistent with GOBP analysis, pathway analysis of healthy DPSCs' unique proteins revealed that RNA metabolism, chromosomal processing, telomere maintenance, growth factor signal transduction, and Janus kinase–signal transducer and activator of transcription (JAK‐STAT) and phosphoinositide 3‐kinase–protein kinase B (PI3K‐AKT) signaling, which are known as ordinary pathways, in both the whole proteome and secretome **(**Figure 3C,D **)**. Identified unique proteins of the decayed group were associated with three dominant pathways, which are cellular stress response, cell cycle, and Nuclear factor kappa B (NF‐κB), in the whole cell proteome. Secretome analysis of the decayed group was very interesting, showing the IGF and Insulin‐like Growth Factor Binding Protein IGFBP signaling, extracellular matrix organization, and senescence‐associated heterochromatic foci (SAHF) pathways **(**Figure 3E,F **)**.

### 3.3 Canonical Pathways and Upstream Analysis Via Ingenuity Pathway Analysis (IPA)

**Figure 4:**
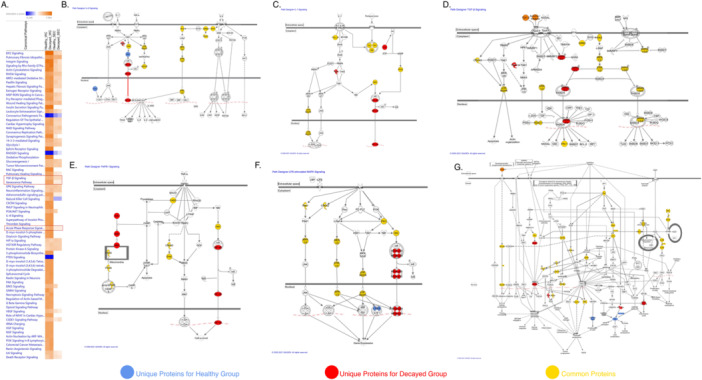
(A) Enriched canonical pathways in whole cell (WC) and secretome (SEC) proteome of both groups. Senescence, TGF‐β Pathways, and Acute Phase Signaling Pathway, which consist of IL‐6, IL‐1, and TNF‐α, were highlighted. (B) IL‐6 Signaling Pathway. (C) IL‐1 Signaling Pathway. (D) TGF‐ β Signaling Pathway. (E) TNFR1 Signaling Pathway. (F) LPS‐Stimulated MAPK Signaling Pathway. (G) Senescence Pathway. In all pathway diagrams, uniquely expressed proteins in the healthy group were represented in blue, uniquely expressed proteins in the decayed group were represented in red, and common proteins were represented in yellow.

We performed canonical pathway analysis via Ingenuity Pathway Analysis (QIAGEN) for every experimental condition that we tested and found a number of pathways that overlap with the proteins in our dataset **(**Figure 4A **)**. When considering the Gene Ontology and previous pathway analysis, we concentrated our attention on several specific pathways about senescence and inflammation. Our evaluation mainly focused on acute‐phase response signaling, which includes IL‐1, IL‐6, and TNF‐α signaling pathways. Canonical pathway analysis revealed that NF‐κB was the common molecule for all of the pathways, and notably, it was identified only in the decayed whole cell proteome dataset **(**Figure 4B,C,E **)**. The presence of NF‐κB in LPS‐induced Mitogen‐Activated Protein Kinase (MAPK) signaling pathway was one of the surprising findings that led us to extrapolate that a microbial product contact occurred in decayed cells, due to the presence of Tool‐like receptor 4 (TLR4) signaling cascade in this canonical pathway. The senescence pathway was the other altered pathway in decayed tooth cells. Interestingly, Retinoblastoma (Rb), which is the key player of cell cycle regulation, was observed only in healthy cells **(**Figure 4G **)**. Furthermore, we observed the presence of TGF‐® in the secretome‐triggered SMAD family member ⅔ (SMAD2/3) signaling in the decayed group DPSCs **(**Figure 4F **)**. When TGF‐® pathway findings were evaluated with the results of IPA upstream analysis, it was observed that TGF‐® activates different signaling cascades in decayed cells, even though it was the top upstream regulator in the secretome of both groups **(**Table 2 **)**.

**Table 2:** Ingeniuty Pathway Analysis Top upstream regulators for each group.

Name	p‐value	Predicted activation
**Healthy whole cell**		
MYC	1.75E‐99	Activated
torin1	4.64E‐80	Inhibited
TP53	1.62E‐77	
LARP1	3.78E‐74	Inhibited
CD437	1.88E‐65	Inhibited
**Healthy secretome**		
TGFB1	1.35E‐45	Activated
MYC	6.16E‐45	Activated
TP53	1.13E‐39	Activated
MYCN	1.63E‐39	
APP	1.86E‐37	
**Decayed whole cell**		
MYC	2.47E‐92	Activated
torin1	1.44E‐77	Inhibited
TP53	3.13E‐76	
LARP1	6.39E‐73	Inhibited
CD437	8.43E‐60	Inhibited
**Decayed secretome**		
TGFB1	9.42E‐43	Activated
MYC	3.03E‐40	Activated
APP	1.30E‐34	
TP53	9.49E‐33	
beta‐estradiol	2.12E‐32	Activated

### 3.4 Label‐Free Quantification (LFQ) Analysis of Common Proteins

The above‐mentioned analysis focused on unique proteins that specifically identified either healthy or decayed tooth DPSCs, and these analyses considered only the presence of molecules in the given dataset. Unique proteins for each group were subjected to Gene Ontology Biological Process and pathway analysis. Differential expression of common proteins among the two groups was also examined via LFQ analysis to gain further insight into the molecular basis of the observed phenotype in the decayed group DPSCs. We compared the expression differences of both cellular proteins and secretome proteins. Detailed lists of differentially expressed proteins were given with log2FC values and p‐values in Supplementary File 1.

**Figure 5:**
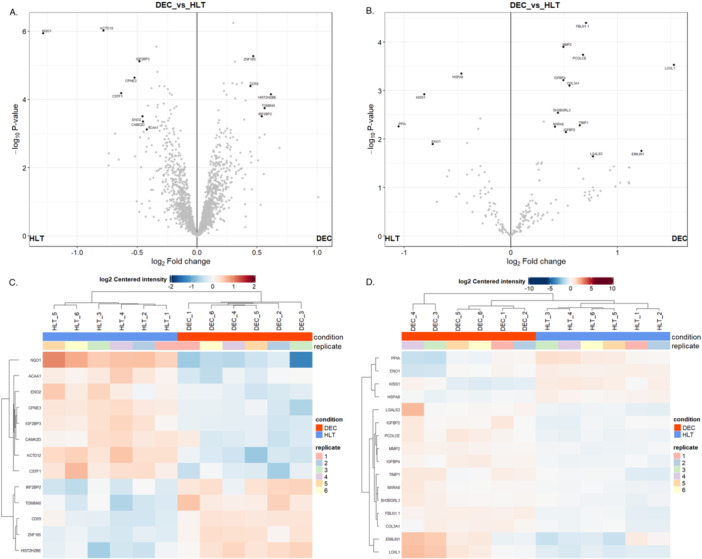
Label‐free Quantification Analysis of the Proteome Dataset. (A) Volcano plot diagram showing the relative expression of whole‐cell proteins between groups. Proteins on the left side of the diagram are downregulated in the decayed group, while those on the right side are upregulated. (B) Volcano plot diagram showing the relative expression of secretome proteins between groups. Proteins on the left side of the diagram are downregulated in the decayed group, while those on the right side are upregulated. (C) Heatmap showing the relative expression of whole‐cell proteins between groups. (D) Heatmap diagram showing the relative expression of secretome proteins between groups.

In comparison of whole cells of the two groups, we found significantly 8 downregulated and 5 upregulated proteins for decayed tooth DPSCs **(**Figure 5A,C **)**. Among downregulated proteins, NAD(P)H:quinone oxidoreductase 1 (NQO1) was associated with detoxification pathways, and it has been known to be involved in biological processes, such as cellular stress response and negative regulation of apoptosis. Potassium channel tetramerization domain containing 12 (KCTD12) and Copine‐3 (CPNE3) were also downregulated in the decayed group, and they have functions in growth factor stimulus. Gamma‐enolase ENO2, which promotes cell survival, particularly in neurons, was one of the downregulated proteins. Downregulation of Insulin‐like growth factor 2 mRNA‐binding protein 3 (IGF2BP3) was remarkable, owing to this, the protein plays an essential role in stemness (CD44 and MYC mediated) via mRNA stabilization and promoting cell adhesion. Upregulated proteins in the whole cell proteome were Zinc finger protein 185 (ZNF185), Cluster of differentiation 59 glycoprotein (CD59), Histone H2B type 2‐E (HIST2H2BE), Mitochondrial import receptor subunit TOM40 homolog (TOMM40), and Interferon regulatory factor 2‐binding protein 2 (IRFBP2). HIST2H2BE was the most remarkable protein among these five proteins because of its involvement in the formation of the antimicrobial barrier and also functioned in antibacterial response. CD59 was another upregulated protein in the decayed group, and that was associated with protein tyrosine kinase signaling mediated T‐Cell activation. Likewise, IRF2BP2, which has functions in B‐cell differentiation, was also upregulated.

In the secretome of the decayed group, we found that 16 proteins were differentially expressed, of which 4 were downregulated and 12 were upregulated **(**Figure 5B,D **)**. Consistent with LFQ results of whole cell proteome, cellular adhesion‐associated proteins KISS1 and Peptidyl‐prolyl cis‐trans isomerase A (PPIA) were downregulated. When examining upregulated proteins in terms of the biological processes, it was observed that many of them have functions associated with extracellular matrix remodeling, which is one of the hallmarks of cellular senescence. Fibulin‐1 (FBLN1.1), Procollagen C‐endopeptidase enhancer 1 (PCOLCE), Lysyl oxidase homolog 1 (LOXL1), Collagen alpha (III) chain (COL3A1), Metalloproteinase inhibitor 1 (TIMP1), Elastin microfibril interfacer 1 (EMILIN1), Matrix remodeling‐associated protein 8 (MXRA8), and Matrix metalloproteinase 2 (MMP2) (72 kDA type IV collagenase) were the upregulated proteins in the decayed group which have roles in ECM organizations. Galectin‐3 (LGALS3), which plays a role in antimicrobial response and T‐cell activation, was also upregulated. Another remarkable protein in our findings was Insulin‐like growth factor binding protein 4 (IGFBP4), known as a major component of SASP and responsible for spreading senescence signaling.

### 3.5 Immunofluorescence Analysis of Inflammatory Markers in Dental Pulp Cells

**Figure 6:**
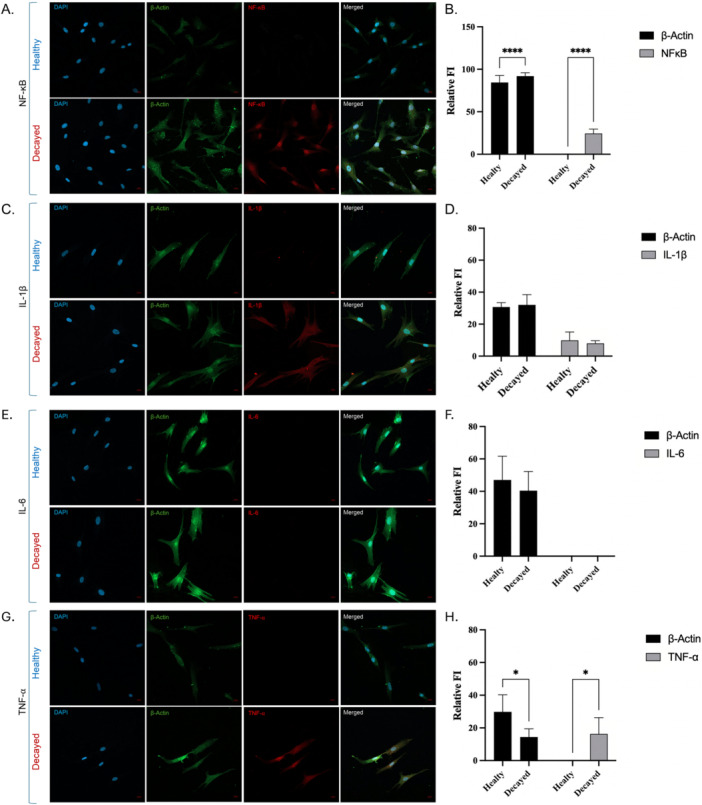
Representative Immunofluorescence Images: Confocal microscopy images showing the expression and localization of pro‐inflammatory markers: (A) NF‐κB, (C) IL‐1β (E) IL‐6, and (G) TNF‐α. Cells were stained for DAPI (blue; nuclei), β‐Actin (green; cytoskeleton), and the respective inflammatory protein (red). The “Merged” column represents the overlay of all three channels. (B) Relative Fluorescence Intensity (FI) comparison for β‐Actin and NF‐κB. Decayed cells exhibit a significantly higher expression of NF‐κB compared to Healthy cells (D) Comparison of IL‐1β and β‐Actin relative FI between Healthy and Decayed groups. No statistically significant difference was observed in IL‐1β levels between the two groups. (F) Fluorescence intensity levels for IL‐6. Both Healthy and Decayed groups show baseline levels of IL‐6 expression without significant divergence. (H) Quantification of TNF‐α relative FI. The Decayed group shows a significant increase in TNF‐α expression compared to the Healthy group.

To deeply investigate the inflammatory pathways provided by the proteomic analysis, we performed immunofluorescence staining for selected inflammatory markers in DPSCs derived from both healthy and carious teeth. Based on the immunofluorescence staining and quantitative analyses a profound inflammatory activation was observed in cells derived from carious teeth. NF‐κB expression was minimal in the healthy group (0.07 ± 0.15), whereas it was significantly elevated in the carious group (24.46 ± 5.15;) **(**Figure 6A,B **) (**Table 3A **)**. Cytokines are secreted proteins; therefore intracellular immunofluorescence signals represent the transient intracellular pool prior to secretion and may vary among individual cells. We detected an increased expression of IL‐1β in some individual cells within the carious group; however, quantitative intensity analysis revealed no statistically significant difference between the healthy and carious groups **(**Figure 6C,D **) (**Table 3B **)**. IL‐6 expression was not detectable above the background level in either the healthy or carious groups **(**Figure 6E,F **) (**Table 3 **)**. TNF‐α expression was undetectable in healthy cells, but showed a significant increase in the decayed group (14.00 ± 9.37) (Figure 6G,H) (Table 3D). The immunofluorescence results supported the inflammatory signaling predicted by the proteomic analysis, particularly the activation of the NF‐κB pathway and increased TNF‐α expression in DPSCs derived from carious teeth.

**Table 3:** Immunoflourescent Staining Results.

	Healthy	Decayed
A	NF‐κB intensity	NF‐κB intensity
	0,07 ( ± 0,15)	24,46 ( ± 5,15)
	*p* < 0,0001	*p* < 0,0001
B	IL‐1β intensity	IL‐1β intensity
	9,86 ( ± 5,26)	7,98 ( ± 1,68)
	*p* = 0,5125	*p* = 0,5125
C	IL‐6 intensity	IL‐6 intensity
	0,0 ( ± N/A)	0,0 ( ± N/A)
	*p* = 0,9988	*p* = 0,9988
D	TNF‐α intensity	TNF‐α intensity
	0.0 ( ± N/A)	14.00 ( ± 9,37)
	*p* = 0,0119	*p* = 0,0119

*Note:* A. Relative NF‐κB Intensity. B. Relative IL‐1β Intensity. C. Relative IL‐6 Intensity. D. Relative TNF‐α Intensity.

## 4 Discussion

Dental caries represent one of the primary oral health issues that has adverse effects on individuals' quality of life. While restorative treatments can successfully remove carious lesions and restore the morphological integrity of the tooth, it is hypothesized that the cells within the dental pulp are compromised due to microbial infiltration associated with the carious process (El Karim et al. 2021). The infiltration of released microbial products leads to inflammation in pulp cells, and progressively results in necrosis (Yu and Abbott 2007). Although restorative treatments can successfully eliminate the lesions that have not yet invaded the pulp, thereby preserving the tooth anatomy, the stress resulting from inflammation initiated by microbial products may still exert detrimental effects on pulp cells (Bergenholtz 2000). Even in non‐necrotic tissues, inflammatory stress can alter cellular function by activating pathways such as pyroptosis, apoptosis, and necrosis in pulp cells of carious teeth (Loreto et al. 2015; Sattari et al. 2022). Under such conditions, apoptotic cells are efficiently removed by immune clearance, and necrotic tissue is typically eliminated during restorative procedures. When the affected tissue is neither apoptotic nor necrotic, the carious lesion is generally managed by restoring the enamel to seal the lesion. Nevertheless, even under aforementioned conditions, we postulate that exposure of pulp cells to infiltrating microbial products or to the inflammatory responses of other cells in close vicinity may induce degenerations in their biology and functions. In the present study, we hypothesize that, in teeth with carious lesions where not reaching the pulp, DPSCs are affected by cellular senescence — a degenerative process characterized by the arrest of cell proliferation while preserving metabolic and inflammatory activity.

To determine whether the carious microenvironment affects DPSC biology, we examined senescence, apoptosis, and cell cycle profiles of cells derived from healthy and carious teeth. Both qualitative and quantitative senescence assays demonstrated that the DPSC populations isolated from carious dental pulps exhibited approximately 20% higher levels of senescent cells compared to those derived from healthy pulps. In line with the senescence assay results, cell cycle analysis revealed a significant shift toward G0/G1 arrest in the carious DPSC population. In parallel, reduced proliferative capacity was observed in these cells, further supporting the presence of a senescent phenotype. Morphometric analysis also showed enlargement of both cytoplasmic and nuclear areas in DPSCs derived from carious teeth, a characteristic feature of senescent cells. Additionally, elevated expression levels of the senescence‐associated markers p16 and p21 were measured in this group. All of these indications suggesting that our initial hypothesis of DPSCs exposed to the carious microenvironment undergo senescence‐associated alterations. Sattari et al (Sattari et al. 2022). demonstrated that exposure of DPSCs to LPS, a microbial product, led to the overexpression of genes implicated in the regulation of the cellular senescence mechanism. In another study, researchers repeatedly exposed DPSCs to LPS and subsequently observed the induction of a senescent phenotype following the onset of inflammatory responses (Feng et al. 2014). Although senescence and apoptosis are distinct cellular processes that share overlapping molecular pathways, senescent cells are typically resistant to apoptosis, as highlighted by Kirkland and Tchkonia (Kirkland and Tchkonia 2017). Nevertheless, senescent and apoptotic cells can coexist within the same tissue microenvironment. Consistent with this notion, we observed that DPSCs derived from carious teeth not only exhibited increased senescence but also showed a ~ 10% higher proportion of apoptotic cells compared to healthy controls. These results align with previous reports demonstrating that pulp tissue cells, including odontoblasts, undergo apoptosis in response to carious insults, contributing to pulp tissue degeneration (Loreto et al. 2015; Wang et al. 2016). These findings suggest that microbial product stress in decayed teeth may simultaneously promote degenerative mechanism, including both cellular senescence and apoptosis, within the dental pulp niche.

After gathering information from the phenotypic properties of the cells, we performed LC‐MS/MS‐based shotgun proteomic analyses to further investigate the molecular basis that underlying the biological alterations observed in decayed tooth pulp stem cells. Comparative proteomic profiling revealed a distinct set of proteins uniquely expressed in the decayed group. Strikingly, both the whole‐cell proteome and the secretome of decayed tooth DPSCs were enriched with molecules associated with inflammation and cellular senescence. In the whole‐cell proteome, ontologies related to catabolic processes, cellular stress responses, and antigen presentation were prominently enriched. In contrast, the secretome was predominantly composed of proteins associated with extracellular matrix remodeling. Reactome pathway analysis further revealed that decayed DPSCs expressed proteins linked to inflammatory response pathways, particularly those orchestrated by NF‐κB signaling. Additionally, molecules involved in cell cycle checkpoint regulation were identified within the dataset. Taken together, these findings suggest that DPSCs derived from carious teeth exhibit a molecular signature characterized by cellular stress, inflammatory activation, and senescence‐related pathways, which may influence neighboring cells within the pulpal niche and impair the regenerative capacity of these stem cells.

The NF‐κB signaling pathway plays a central role in orchestrating the inflammatory responses associated with numerous oral diseases, including dental caries and periodontitis (Chen et al. 2024). It is also mentioned that NF‐κB signaling has a pivotal role in response to pathogen induced inflammation. In parallel with our findings, the involvement of this pathway in DPSC odontoblast differentiation was also reported (He et al. 2014). Upregulation of NF‐κB may also support the odontoblastic differentiation of DPSCs as a defensive mechanism of pulp against microbial contact (Zhu et al. 2019; Chen et al. 2021; Long et al. 2024). Examination of the secretome dataset of decayed tooth DPSCs revealed the presence of Bone Morphogenetic Protein (BMP), a key regulator of odontoblast differentiation and essential for dentin formation (Liu et al. 2022). Combined interpretation of the whole‐cell proteome and secretome data points to a prominent role of NF‐κB signaling, implicating this pathway in both inflammatory responses and odontoblast differentiation. Enrichment of IGF and IGFBP signaling‐related ontologies, along with the expression of ECM remodeling proteins, was evident for cellular senescence in the secretome dataset. The presence of IGFBPs and ECM remodeling molecules is an indicator of active senescence mechanisms in these cells. These pathways and biological processes are recognized components of the senescence program and are the hallmarks of SASP (Severino et al. 2013; Acar et al. 2020; Ayaz‐Guner et al. 2020).

Following the biological process and pathway analyses, we conducted canonical pathway analyses using the Ingenuity Pathway Analysis (IPA) to further explore the signaling pathways that related to observed cellular phenotype. Since the Reactome analysis indicated activation of the NF‐κB signaling pathway, we specifically examined signaling cascades involving IL‐1, IL‐6, and TNF‐α — key molecules of the Acute Phase Stress Response, a primary defense mechanism against microbial challenge (Yu et al. 2021). Our dataset revealed that the NF‐κB transcription factor, detected exclusively in the proteome of decayed DPSCs, was involved in the activation of all three pathways. Canonical pathway analysis further demonstrated that the inflammatory factors expressed by decayed DPSCs possess the capacity to trigger inflammation in neighboring niche cells and to exacerbate chronic inflammatory processes. Importantly, the resulting inflammation may not just be restricted to the pulp tissue alone but could potentially affect systemic physiology. Moreover, canonical pathway analysis showed that the NF‐κB transcription factor, specifically the p65‐p50 heterodimer, plays a critical role in regulating the LPS‐stimulated MAPK signaling pathway. To evaluate the inflammatory response in DPSCs of decayed group, the expression of NF‐κB, IL‐1β, IL‐6, and TNF‐α were assessed by immunofluorescence staining. Consistent with the pathway analysis, increased expression of NF‐κB and TNF‐α was observed in DPSCs derived from carious teeth. Whereas no significant differences were detected in the intracellular expression levels of IL‐1β and IL‐6 between the groups. Given that interleukins are primarily secreted proteins, the absence of differences in their intracellular staining was expected.

Previous studies demonstrated the central importance of NF‐κB in pathogen induced MAPK signaling via TLR4, emphasizing its pivotal role in the early host inflammatory response (Du et al. 2010; Nyati et al. 2017). In aligned with the study by de Farias et al (de Farias et al. 2024), our study demonstrated that cellular senescence in dental pulp cells leads to morphological enlargement of cells together with reduced proliferative and an enhanced pro‐inflammatory profile, highlighting the detrimental impact of senescence on pulp tissue homeostasis. Considering that LPS stimulation was shown to promote cellular senescence (Suzuki et al. 2019, 2022), it is plausible to propose that DPSCs from decayed teeth may undergo senescence through activation of the TLR4‐MAPK‐NF‐κB axis following the microbial product contact.

In addition to the proteins uniquely identified in each group, we conducted LFQ analyses to investigate proteins commonly expressed in both groups with a differential expression level. Comparative LFQ analysis of the whole cell proteomes revealed that several proteins downregulated in DPSCs derived from carious teeth, relative to the control group, are implicated in the regulation of stemness and apoptosis. The loss of stem cell properties in DPSCs under stress is a well‐acknowledged phenomenon (Zhu et al. 2019; Çankirili et al. 2020; Iliopoulos et al. 2022). Furthermore, we propose that changes in the expression of proteins involved in apoptosis regulation may reflect a shift along the senescence‐apoptosis axis. Notably, the NQO1 protein, which was found to be downregulated, is a component of the Keap1–Nrf2 signaling pathway, which has been shown to play a critical role in the senescence–apoptosis switch mechanism (Kopacz et al. 2020).

Additionally, both the whole‐cell proteome and the secretome exhibit reduced expression of proteins associated with cell adhesion. At this stage, it is relevant to consider these findings alongside the differential expression of CD146—a surface adhesion glycoprotein used during cell characterization (Matsui et al. 2018). The decreased expression of IGF2BP3 and KISS1, coupled with a lower proportion of CD146‐positive cells in DPSCs from carious teeth compared to the control group, collectively indicate an impaired adhesion capacity, which may explain the pulp recession commonly observed in the progression of dental caries.

The degradation of the ECM undoubtedly plays a key role in the loss of adhesion capacity in dental pulp cells. Our analysis of the proteins upregulated in the secretome of carious tooth‐derived DPSCs revealed that a significant proportion of these are ECM‐modifying proteins. Notably, proteins such as LOXL1, TIMP1, and MMP2—found to be upregulated in the secretome—are well‐established contributors to ECM remodeling (Mavrogonatou et al. 2019). ECM remodeling is a hallmark of cellular senescence and proteases such as collagenase, a major component of the SASP, involved in this process (Lopes‐Paciencia et al. 2019; Vamvakas et al. 2017). Considering the senescent phenotype of DPSCs derived from carious teeth, the overexpression of ECM remodeling molecules in their secretome can be linked directly to the observed loss of cell adhesion. Another notable finding was the increased expression of IGFBP4, a protein previously identified as an important component of the SASP and implicated in the propagation of senescence‐associated signaling (Severino et al. 2013; Acar et al. 2020). The presence of IGFBP4 in the secretome suggests that senescent DPSCs may influence neighboring cells within the pulpal niche through paracrine signaling.

Among the upregulated proteins in DPSCs from decayed teeth—both in the whole‐cell and secretome profiles—were LGALS and IRF2BP2, which are involved in the T‐ Cell activation and B lymphocyte differentiation. These findings are in line with previous studies showing that cellular senescence in dental pulp cells is closely associated with alterations in immune regulation and inflammatory signaling, promoting a pro‐inflammatory microenvironment (da Silva et al. 2025). Moreover, HIST2H2BE, with an increased expression in the whole proteome, was previously identified as a contributor to antimicrobial barrier formation (Tollin et al. 2003; Agak et al. 2021). Collectively, these findings suggest that even in the absence of direct exposure, pulp cells were responsive to pathogen‐originated products diffusing through dentin. The observed upregulation of immune and antimicrobial effectors indicates an active response by the cells to potential bacterial invasion.

## 5 Conclusion

**Figure 7:**
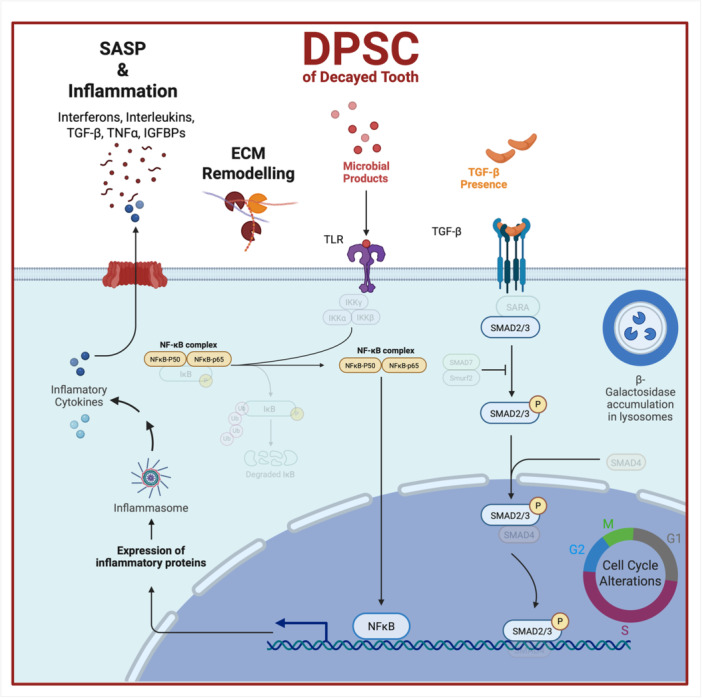
It is hypothesized that microbial products derived from dental caries infiltrate the dental pulp tissue and exert their effects on dental pulp stem cells (DPSCs) primarily through the activation of Toll‐like receptors (TLRs). This interaction leads to the induction of cellular senescence and the initiation of an inflammatory response, predominantly mediated via the NF‐κB signaling pathway. As a hallmark of the senescence‐associated secretory phenotype (SASP), various pro‐inflammatory cytokines and matrix‐remodeling enzymes are secreted into the extracellular milieu, thereby contributing to both sustained inflammation and extracellular matrix (ECM) remodeling within the pulp niche. Furthermore, the upregulation of transforming growth factor‐beta (TGF‐β), as part of the SASP response, results in the activation of the SMAD signaling pathway, which may influence the behavior and fate of neighboring cells in the microenvironment.

In the treatment of dental caries that have not reached the pulp chamber, restorative approaches generally help conserve the vitality of the cells within the pulp and preserve the tooth. However, the impact of caries‐associated stress on pulp cell biology has yet to be fully elucidated. In this study, we examined the biological and molecular properties of DPSCs from teeth with caries that had not yet reached the pulp chamber. Our findings revealed that even when caries do not reach to the pulp, DPSCs are still affected by this stress and undergo cellular senescence. The induction of the senescence process—an irreversible mechanism—triggers chronic inflammation in DPSCs. Furthermore, senescent DPSCs secrete SASP factors that may have degenerative effects on other tissues in the body **(**Figure 7 **)**. Considering their anatomical location and secretory activity, it is conceivable that senescent DPSCs may contribute to local tissue alterations and potentially influence broader inflammatory processes. Finally, our study suggested that there is a significant and non‐negligible link between dental caries and DPSC senescence. To alleviate the harmful systemic burden induced by DPSCs that have entered senescence due to caries, it is crucial to develop regenerative strategies that combine restorative treatments with senotherapeutic approaches.

## 6 Limitations

Limitation of this study came from the relatively small number of donor samples and the use of pooled cell populations for downstream analyses. Cells obtained from eight donors in each group were pooled to minimize inter‐individual variability and to obtain sufficient material for comprehensive proteomic analyses. While this approach allows the identification of common molecular patterns associated with the studied condition, it may also mask donor‐specific variations. Therefore, future studies including larger sample sizes and individual‐level analyses will be important to further validate and expand upon the findings reported here.

## Author Contributions

**Sebahat Melike Durukan:** conceptualization, data curation, formal analysis, investigation, visualization. **Mustafa Burak Acar:** conceptualization, formal analysis, project administration, supervision, writing – original draft. **Banu Çiçek Tez:** conceptualization, data curation, visualization, writing – original draft. **Ahmet Şimşek:** investigation, methodology, visualization. **Sura Hilal Ahmed Al‐Sammarrie:** investigation, methodology, visualization. **Zeynep Günaydın:** methodology, investigation visualization. **Melis Güzel:** methodology, investigation. **Başak Bilge Süer:** methodology, investigation. **Şerife Ayaz Güner:** conceptualization, supervision, writing – review and editing. **Hüseyin Güner:** methodology, investigation, visualization. **Nicola Alessio:** project administration, supervision, writing – review and editing. **Kemal Erdem Başaran:** methodology, investigation, visualization. **Zeynep Burçin Gönen:** project administration, resources, supervision, validation, writing – review and editing. **Servet Özcan:** funding acquisition, project administration, resources, supervision, validation, writing – review and editing.

## Ethics Statement

The study was approved by Erciyes University Clinical Research the Ethics Committee of (No: 2019/573, Date: July 24, 2019). The research has been carried out in accordance with the World Medical Association Declaration of Helsinki and that all subjects provided written informed consent.

## Conflicts of Interest

The authors declare no conflicts of interest.

## Supporting information


**Supplementary File:** jcp70172‐sup‐0001‐Supplementary_File_1.xlsx.

## Data Availability

All MS data has been submitted to the ProteomeXchange Consortium via the PRIDE partner repository with the dataset identifier, PXD064565. Reviewers can access the dataset using token “0rJWFQZeaCol”. Alternatively, reviewers may access the dataset by logging in to the PRIDE website using the following account details: Username: reviewer_pxd064565@ebi. ac. uk, Password: rcVcethMG0CW.
